# Potential Diagnostic Systems for Coronavirus Detection: a Critical Review

**DOI:** 10.1186/s12575-020-00134-4

**Published:** 2020-09-01

**Authors:** Elena Ekrami, Mahdi Pouresmaieli, Fatemeh Barati, Sahar Asghari, Farzad Ramezani Ziarani, Parvin Shariati, Matin Mamoudifard

**Affiliations:** 1 Department of Industrial and Environmental Biotechnology, National Institute of Genetic Engineering and Biotechnology (NIGEB), Tehran, Iran; 2 Department of Microbiology, School of Medicine, Golestan University of Medical Sciences, Gorgan, Iran

**Keywords:** SARS-CoV-2, Detection, Biosensor, RT-PCR, Immunoassay, Nano-materials

## Abstract

**Abstract:**

Currently there are no effective anti-viral drugs for SARS-CoV-2, so the primary line of defense is to detect infected cases as soon as possible. The high rate of contagion for this virus and the highly nonspecific symptoms of the disease (Coronovirus disease 2019, (Covid-19)) that it causes, such as respiratory symptoms, cough, dyspnea, fever, and viral pneumonia, require the urgent establishment of precise and fast diagnostic tests to verify suspected cases, screen patients, and conduct virus surveillance. Nowadays, several virus detection methods are available for viral diseases, which act on specific properties of each virus or virus family, therefore, further investigations and trials are needed to find a highly efficient and accurate detection method to detect and prevent the outcomes of the disease. Hence, there is an urgent need for more and precise studies in this field. In this review, we discussed the properties of a new generation of coronaviruses (SARS-CoV-2) following routine virus detection methods and proposed new strategies and the use of potential samples for SARS-CoV-2 detection.

**Graphical Abstract:**

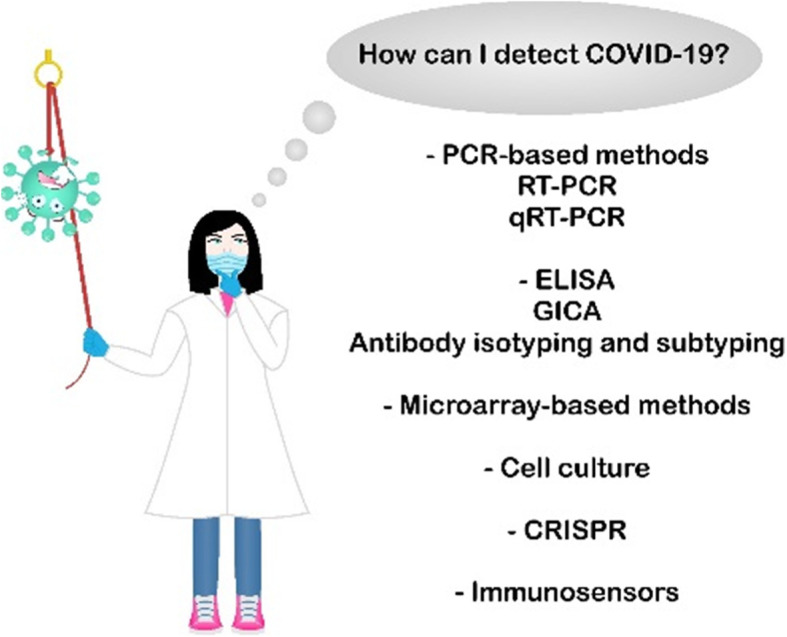

## Introduction

On 30th January 2020 the WHO declared a global “public health emergency of international concern” (PHEIC) regarding the epidemic caused by the 2019 novel coronavirus (2019-nCoV), which started in a Wuhan seafood market, Hubei province [1]. This virus is genetically very similar to the bat SARS-CoV in the subgenus *Sarbecovirus* [2]. The WHO has currently named the disease, which has spread so rapidly throughout the world, as coronavirus disease 2019 (COVID-19) [3]. The 2019 novel coronavirus (2019-nCoV) has also been renamed as severe acute respiratory syndrome coronavirus-2 (SARS-CoV-2) by the International Committee on Taxonomy of Viruses. Coronaviruses got their name from their morphology when observed under a microscope. The virus consists of a core of genetic material surrounded by an envelope with protein spikes. This gives it the appearance of a crown. The word Corona means “crown” in Latin. Coronaviruses are zoonotic [4] meaning that the viruses are transmitted between animals and humans. Coronaviruses can be classified into four genera (α, β, γ, and δ), and these viruses are detected in a very wide selection of animal species, including humans [5]. The virus that causes the COVID-19 disease belongs to the β-coronavirus genus. Since the beginning of the twenty-first century, three kinds of coronaviruses have crossed the species barrier, causing deadly pneumonia in humans. These include the severe acute respiratory syndrome SARS-CoV [6], Middle-East respiratory syndrome (MERS-CoV) [7], and SARS-CoV-2 [8] coronaviruses. Unlike previous coronaviruses that caused large-scale epidemics such as the Middle East Respiratory Syndrome (MERS) and Severe Acute Respiratory Syndrome (SARS), the transmission rate for SARS-CoV-2 is much higher, with an average of two to three people becoming infected for every already infected person [9]. SARS-CoV-2 is mainly transmitted to healthy persons by exposure to the respiratory droplets of one infected person through close interactions. After entering the body, this virus is first observed in the nasal mucosa and mouth, and is then transferred to the lungs of the exposed individuals. People infected with SARS-CoV2 will experience weak symptoms, but in susceptible persons with certain underlying diseases like diabetes, hypertension, heart and autoimmune diseases, severe respiratory and lethal sickness or damage to some other vital organs such as the gastrointestinal tract, liver, kidney or the central nervous systems will take place [10].

After entrance to the body, the virus finds its target cells through its receptor, namely angiotensin-converting enzyme 2 (ACE2). ACE2 is mainly expressed on the epithelial cells of healthy lung tissue. But, it is reported that other types of cells may also present ACE2 thus making them vulnerable to the virus [11]. Besides, it has been also reported that SARS-CoV-2 is indirectly harmful to immune cells, mostly T cells, and macrophages, eliciting their destruction. In this regard, there are some COVID-19 immunopathogenesis factors including granulocyte colony-stimulating factor (G-CSF), tumor necrosis factor-α (TNF-α), macrophage inflammatory protein, (MIP-1A), monocyte chemoattractant protein-1 (MCP-1), interferon gamma-induced protein-10 (IP-10), interleukin-1 (IL-1), IL-2, IL-6, IL-7, and IL-10 that can be used as detection markers in COVID-19 patients [12].

Because of the high rate of contagion and the highly nonspecific symptoms of the COVID-19 disease, such as respiratory symptoms, cough, dyspnea, fever and viral pneumonia [8], the establishment of precise, fast and inexpensive diagnostic tests methods that can lower the risk of spreading infection, alleviate the strain on the healthcare system, and mitigate the cost of care for both individuals and the government, can improve the health care system to combat this and similar diseases. Generally, for the detection of viral diseases like COVID-19, different kinds of diagnostic strategies will be purposed including: inspection of physical symptoms, clinical evidence, radiological images and para-clinical findings. Para-clinical detection methods are also categorized as nucleic acid amplification tests (NAATs), viral sequencing, serological assays, and viral culture and real-time polymerase chain reaction (RT-PCR) assays.

The main goal of the current review is to provide a comprehensive overview on the newly developed methods and also potential techniques for the detection of viral diseases especially corona ones to help researchers around the world assess the advantages and disadvantages of each method and select the right one to efficiently control such diseases. Before starting, a brief overview of viruses and their types and properties is presented.

## Virus Structure and Function

Viruses are small infectious agents and obligatory intracellular parasites that use the host cell’s biosynthetic and metabolic machinery to survive and replicate their genomes, and in doing so, they cause damage and subsequent destruction of host cells, which ultimately leads to different types of diseases in humans.

Viruses have different types of structures that include genomes comprised of single or double-stranded DNA or RNA. The genome is encircled by a protective cover made of a protein known as the capsid, which is encoded by the viral genome. Such proteins that are structurally associated with the nucleic acid are referred to as nucleoproteins [13]. Viruses infect all types of life forms [14], and can use viral digestive enzymes to dissolve the host cell membrane, thereby gaining entry into the cell. However, they do not contain various enzymatic systems apart from digestive enzymes which are used to enter the cell. So, because of their structure and their enzyme system as mentioned above, antibiotics are not effective for viral infections [15]. There are different kinds of viruses that can infect humans, and until now only 219 viral species have been detected. The first type of virus that was discovered in 1901 was the yellow fever virus. Every year new types are found, showing a rise in the number of known viruses that cause various diseases [16]. An example of this is the current novel infectious coronavirus, SARS-CoV-2, which has quickly become widespread throughout the world.

### The History of the SARS-CoV-2 Virus

The *Nidovirales* order, includes *Coronaviridae, Roniviridae,* families and Coronaviruses (CoVs) are the greatest groups of viruses that relate to this order and the *Coronaviridae* family [17]. Coronaviruses have a single-stranded RNA genome, 26 to 32 kilobases in length [18]. They usually have a wide range of hosts to infect, such as amphibians and mammals, but their main hosts for infection are avian [19]. Mammalian hosts include mice, bats, masked palm civets, camels, cats, and dogs [20], but lately, the number of new mammalian hosts infected by coronaviruses is growing at an alarming rate [18]. For example, fatal severe diarrhea syndrome in pigs, associated with an HKU2-related coronavirus of bat origin was reported in 2018 [21]. There are different coronaviruses, but the majority of them are associated with mild clinical symptoms [18]. SARS-CoV-2 is a virus belonging to the *Coronaviridae* family, which causes infection in humans; SARS-CoV, MERS-CoV, and SARS-CoV-2 can cause acute disease, while NL63, HKU1, OC43, and 229E cause mild symptoms [22]. The first time that severe acute respiratory syndrome (SARS) coronavirus (SARS-CoV) appeared, was during November 2002 in Guangdong, southern China, and the first time the Middle East respiratory syndrome (MERS) coronavirus (MERS-CoV) emerged, was in 2012 in Saudi Arabia [7, 23, 24]. During 2002–03, more than 8000 infected cases by SARS-CoV with more than 774 deaths in 37 countries were confirmed. The MERS-CoV outbreak in September 2012, caused 2494 confirmed cases of infection and 858 mortalities, including 38 fatalities in South Korea [25, 26]. In late December 2019, many infected patients with viral pneumonia were reported, but notably, all of them were associated with the Huanan seafood wholesale market [27]. Next-generation sequencing confirmed the presence of a new virus which infected humans, and was provisionally named the 2019 novel coronavirus (2019-nCoV).

From Mar 21, 2020, China has reported 80,967 confirmed cases across the country, with 3248 fatalities [28]. Infections in hospital workers and doctors and families were also revealed, thus indicating that human-to-human transmission of the new virus was possible [29]. Further observation showed that this virus outbreak happens quickly by human-to-human transmission [30]. It started from Wuhan and then spread to other areas, causing more than 44,000 cases of COVID-19 in China until Feb 12, 2020, based on statistical data. Besides, outside China including Hong Kong, Macao, and Taiwan, more than 400 cases were reported in Thailand, Japan, Turkey, Italy, Singapore, France, Australia, and Canada [31]. The number of reported cases was growing appreciably. Clinical manifestations related to this virus infection ranged from no symptom to fatal pneumonia [32]. Most of the patients had a high fever and some had dyspnea, with chest radiographs revealing incursive lesions in the lungs [8].

Reported cases with SARS-CoV-2 are now more prevalent, and as of 10 August 2020, 19.9 M cases have been reported in 203 countries, and the number of death stands at 49,242 in the world [28] at 14:57 GMT. At present, available data shows that SARS-CoV-2 which infected the human population originated from a bat source, at present, available data shows that SARS-CoV-2 which infected the human population originated from a bat source, Pangolins have been cited as possible intermediate between humans and bats.

**Fig. 1:**
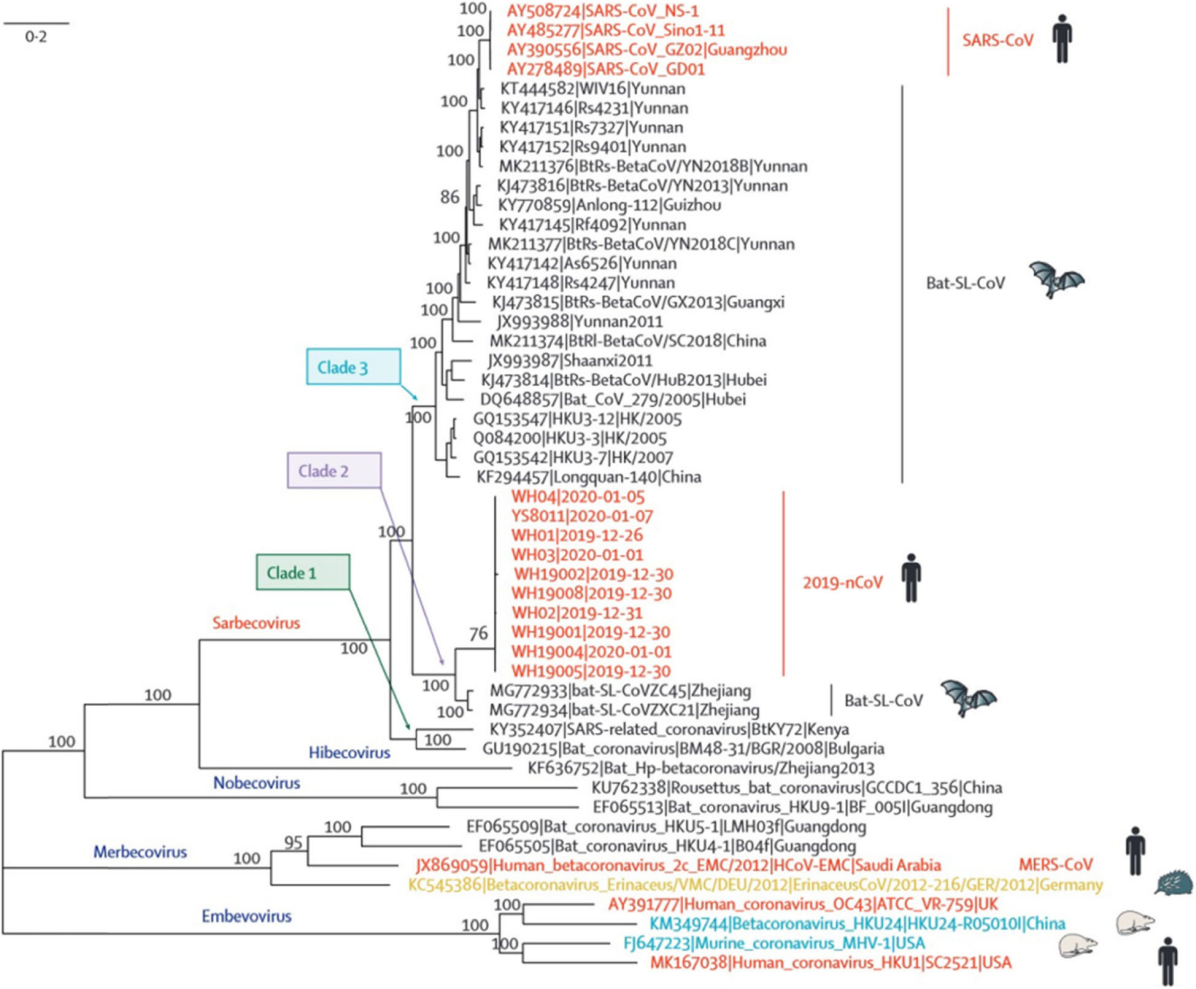
Phylogenetic analysis of full-length genomes of Sars-CoV-2 and representative viruses of the genus beta-coronaviruses (reprint with permission from ref. [33]

Full-genome sequencing of 2019-nCoV shows that this virus is genetically similar to the bat-SL-CoVZC45 (sequence identity 87・99%; query coverage 99%) and SARS-like betacoronavirus of bat origin, bat-SL-CoVZXC21 (accession number MG772934;23 87・23%; query coverage 98%) **(**Fig. 1**).**

Genetic-wise, the SARS-CoV-2 strains are less similar to SARS-CoV and MERS-CoV (about 79 and 52%, respectively). Sequencing of samples from China especially Wuhan indicated that this virus comes from the subgenus *Sarbecovirus*. Also, it shows that it is more analogous to the bat-SL-CoVZC45 and bat-SL-CoVZXC21 viruses, both of which are bat-derived coronavirus strains, than to other recognized human-infecting coronaviruses, which caused the SARS outbreak of 2003 [34]. Coronaviruses have an evolutionary rate of 10^− 4^ nucleotide substitutions per site per year [18]. Their genetic sequence endures mutations at every replication cycle. The facts and information available so far indicate that SARS-CoV-2 emerged from one source instantaneously. However, as the virus is transmitted to more individuals, arising mutations should be investigated. All the evidence indicates that there is a relationship between bats and the novel coronaviruses, but other observations also show that another animal may act as an intermediate.

First, primary reported cases of coronavirus were in late December 2019, but at this time most of the bats in Wuhan were in hibernation. Second, as mentioned before, the first reported cases were from the Huanan seafood market, but facts show that there are not any bats at this market. However, other non-aquatic animals (like mammals) were accessible for sale. Third, genetic-wise, the detected sequence of 2019-nCoV has less than 90% similarity to the bat-SL-CoVZXC21 and bat-SL-CoVZC45 viruses. This data indicates that 2019-nCoV’s direct progenitor is not bat-SL-CoVZC45 and bat-SL-CoVZXC21 [35]. Fourth, other animals such as the masked palm civet for SARS-CoV and dromedary camels for MERS-CoV were an intermediate host for both SARS-CoV and MERS-CoV, and in both SARS-CoV and MERS-CoV, bats were acting as natural source and humans were final hosts [36, 37] So, with these facts and information, it shows that the SARS-CoV-2 virus causing the Wuhan outbreak might have also started by bats, and it might have been transmitted to humans through another unfamiliar animal(s) which was accessible and available for sale at the Huanan seafood wholesale market.

## Methods for Virus Detection

There are many techniques for the detection of viruses, the functions of which depend on virus type and virus particle properties. Herein, we review the most important types.

### Immunofluorescence Methods

Immunofluorescence (IF) staining including the “indirect” fluorescent-antibody assay (IFA) are classified as fast methods for the detection of viral antigens, which typically take at least 2 h or more to complete. These techniques have ideal specificity and sensitivity [38].

In other words, they are based on enzyme immunoassays (EIAs) techniques like the enzyme-linked immunosorbent assay (ELISA) and make use of similar protocols and materials, such as monoclonal antibodies. Therefore, ELISA has become the most useful assay for simple and sensitive virus testing due to its speed, ease of operation, and easy interpretation of results [39].

Despite the many benefits of this method, there are also disadvantages, which limit its use to detect all viruses. The most important limitation of ELISA is the time-consuming antisera production, which must be conducted in specialized laboratories. The main differences between ELISA and nucleic acid-based detection methods are accessibility and low costs [40].

On the other hand, the ELISA method can detect viral antigens and proteins of the recombinant virus using antisera. Generally, this strategy is efficient and sensitive, but in many situations, for example, where identification of specific viral species/strains is required, ELISA is often not appropriate [41].

Therefore, ELISA does not have sufficient flexibility and compatibility, which is an intrinsic property in some molecular methods.

### Nucleic Acid Amplification Tests (NAATs)

Since the early twentieth century, the detection of viruses in clinical samples via molecular methods has become widespread. Diagnosis of viruses can be done directly using clinical samples and cell culture supernatants via specific nucleic acid probes, which can find their complementary target viral RNA or DNA sequences or by using NAATs [42]. Also, purification and separation of nucleic acids from antibodies is easy, which is not always the case for viral antigens, whose detection may be inhibited by antigen/antibody complexes [43].

While methods based on nucleic acids are functional for most viroids and some viruses, the technique that has been most successfully exploited is based on the Polymerase Chain Reaction (PCR).

#### Polymerase Chain Reaction (PCR)

The diagnosis of viruses through PCR methods was established in the early 1990s [44]. In this technique, many copies of one DNA template are made using primers, enzymes, and variable temperatures [45]. Unfortunately, the small amounts of DNA liberated into the laboratory environment through the opening of the tubes after thermal cycling could eventually be detected by the PCR method, resulting in reporting false-positive results. To solve these problems, closed-tube and homogeneous PCR assays-commonly known as real-time PCR or quantitative PCR (q-PCR) are used [46]. This technique is quickly performed for diagnostic applications due to the generated fluorescent signal, which could be detected at a time (‘real-time’) or the end of the process (‘endpoint’) without the contamination risk. Initially, Real-time PCR for virus detection was developed to increase sensitivity [33], thus playing a critical role where antibody methods could not be used [47].

Real-time PCR methods have been established for three important targets. 1. Real-time PCR is quicker than ELISA to establish, considering the development of antibodies for new viruses, 2. This method is more functional, especially in routine laboratories, 3. Real-time PCR is more cost-effective due as compared to the expensive antibody production for immunosorbent assays. Thus, it seems that real-time PCR enables high-throughput testing at a relatively low per-sample cost and great speed to set up a new assay [48].

#### Isothermal Amplification

The main theory of isothermal amplification of DNA is separating two strands of the template through non-thermal ways, such as helicase dependent amplification (HDA) [49] and Recombinase Polymerase Amplification (RPA) [50].

Helicase dependent amplification (HDA) uses a helicase to separate the strands of double-stranded DNA leading to primer annealing and extension of the supplementary strand via DNA polymerase enzyme at 65 °C. Reaction times for HDA are generally in the range 30–90 min. The function of this method relates to the short products of nearly 70–120 bp [49].

Recombinase Polymerase Amplification (RPA) uses a recombinase to produce a complex with primers resulting in extension without thermal denaturation. The reaction is carried at a low reaction temperature (between 37 and 42 °C), which can easily be provided via a low power source. However, the production of non-specific amplification pieces is common due to the low reaction temperature. Nevertheless, RPA has a short reaction time, which is typically < 30 min [50].

An alternative isothermal amplification approach is to design primers such that the extension products contain single-stranded primer binding sites [51]. Accordingly, loop-mediated isothermal amplification (LAMP) is the most ordinarily used method, using three pairs of primers (internal, external and loop primers), to get an amplification product which has single-stranded loop regions to which primers can bind without template denaturation [52] at a reaction temperature of around 65 °C. The supplementary sequences of internal primers can stick to themselves to produce loop structures, while the extension of the external primers leads to the transposition of the extension products of the internal primers. The products of LAMP reactions are interchangeably oriented repeats of the target sequence. The addition of loop primers increases amplification due to the priming role of loops. These regions can raise sensitivity and reduce reaction times, which are desired outcomes.

Loop-mediated isothermal amplification (LAMP) can be modified to detect RNA targets by the addition of reverse transcriptase enzyme to the reaction. Besides, in RT-LAMP, reverse transcription and amplification of cDNA continue temporarily at a single temperature of around 65 °C.

Now, there are detection kits produced via this approach for a wide range of pathogens including bacteria and viruses. The mix of LAMP and innovative microfluidic equipment including Lab-on-a-chip can lead to design genetic checkpoint testing systems [51, 53].

As an example, this technique has been used for the diagnosis of the Zika virus with high sensitivity. This cost-effectiveness system can be freeze-dried for distribution and replace reverse transcription-quantitative polymerase chain reaction (RT-qPCR) [54].

#### Next-Generation Sequencing (NGS)

Next-generation sequencing (NGS) has contributed to the field of virology through the identification of new viruses via popular platforms such as pyrosequencing [55].

NCBI BLAST and PLAN are the most popular Blast tools but they have a limitation, and that is the acceptance of a limited number of sequences in the flat fasta format. On the other hand, Galaxy is more flexible with NGS data but not for novel virus discovery purposes. In general, map NGS reads the human genome and eliminates host reads after the Blast steps. The remaining data are analyzed to classify into non-human, microbial, or viral integrated sequences. This approach can make a difference in costs, ease of sequence assembly, and identification, the key distinctions lie in the nucleic acid purification techniques employed [56]. In one study, Czotter et al. [57] used virus-derived small RNAs. Through their deep sequencing, they can determine the viromes of vineyards in Hungary. NGS of these RNAs, enabled them to diagnose routine viruses and new ones, which had never been described in Hungary before.

### Electron Microscopy (EM)

Electron microscopy (EM) is one of the primary virus detection methods, especially in unsuspected and unknown agents. This tool can capture live images of cells and tissues with high-resolution. In most cases, the observed morphology can lead to immediate detection at the family level based on particle size, shape, and stability. Human viruses include two major morphological categories, naked or enveloped. Naked viruses are icosahedral; with rigid capsids that can endure drying processes and keep their spherical structure in negative stains. Enveloped viruses have an extra outer cover that is usually derived from host membranes. These viruses (except for poxviruses) may show various shapes [58].

Transmission electron microscopy (TEM) is a good primary step in virus diagnosis, which targets proteins within the virus structure and is harmless to RNA or DNA genomes. Immuno-electron microscopy (IEM) is also based on the same serological principles as ELISA and can be a useful approach for more virus identification [59].

### Cell Culture

The use of human cells instead of embryonated eggs and laboratory animals, for in vitro isolation of viruses was first carried out in the 1900s. It has been improved since the 1970s due to the availability of highly purified reagents and commercially-prepared cell lines [60]. The progress in virus isolation using cultured cells has occurred since the addition of antibiotics to the cell culture media, development of chemically defined culture media, and the use of cell-dispensing equipment for preparing replicate cultures [61].

Cell cultures provide large numbers of cells as hosts for the virus and help decrease the use of experimental animals, and hence a lower risk of contamination. Therefore, viruses reach high titers in the cells which are accessible for microscopic examinations. Also, cell cultures are less expensive than eggs and animals [42].

### Nanoelectromechanical Devices

High-frequency nanoelectromechanical systems (NEMS) [62] are being considered as new sensors and devices. It has been proved that the selective molecular binding to the surface of nanomechanical oscillators may lead to detecting pathogen viral binding through observing their effects on the natural frequency shift of NEMS devices [63].

This method for virus detection is still in its infancy and needs further development to be more efficient. Additional information will be mentioned in the next chapter.

### Use of Electrochemical and Nanobiosensors to Detect Virus-Infections

#### Electrochemical Studies

In typical methods for diagnosing viruses, instruments and staff are needed, also, the detection of viruses is time-consuming.

Applying nanoparticles (NPs) in combination with electrochemical detection is promising in detecting viruses. A biosensor is an analytical tool applied for the detection of analytes that combine a biological component with a physicochemical detector [64].

Electrochemical nanosensors have advantages that include being quick, precise, selective, practical, and economical when compared to traditional techniques. The NP-based biosensor is better for detecting pathogenic microorganisms in clinical samples because they are user-friendly and have high specificity, and low costs [65–67].

#### NanoBiosensor

Analyses of biosensor-based researches have been very significant in the last three decades, due to their advantages that include low costs, quick reactions and responses, easy to use, and being user- friendly [68]. These devices detect the existence or concentration of a biological analyte, like a biological system or a microorganism, or a biomolecule [69]. Typically biosensors consist of three components: a part which recognizes the analyte or biological identification part, a signal transducer, and an amplifier or a part which is known as the reader device [70]. A new achievement in biosensor technology is the immobilization of biologically sensitive substances on the surface of a biosensor. Bioreceptor bases are commonly considered as biomarkers, nucleic acids, enzymes, microorganisms, tissues, viruses, bacteria, and antigens. Electrochemical, various field-effect transistor-based methods, and optical methods are defined as the most popular traditional types of biosensors [71].

Nanomaterials (NMs) developed new possibilities for the expansion of electrochemical biosensors [72, 73]. Nanomaterials (NMs) in combination with new advances in biosensor structures help to provide developed novel electrochemical assays. These materials have great advantages because of promoting electron transfer reactions, electrical conductivity, great surface area, mechanical robustness, and good chemical stability [74]. The electrochemical nanobiosensors have been applied to cancer diagnostics and detection of infectious microorganisms, such as viruses, etc. [75].

Production of biosensors, applying the techniques of electrochemical impedance spectroscopy (EIS) CV, and square wave voltammetry (SWV), helps fast biosensing for several kinds of analytes. Electrochemical impedance spectroscopy (EIS) is applied at surface area and interaction modification agents and the electrode surface [76]. Due to the occurrence of an electrochemical reaction at the electrode surface over interaction with the exact molecule, impedance biosensors have been used more in environmental monitoring of disruptive drugs and chemicals, the interaction between antibody and antigen and DNA strains. The EIS provides data regarding surface adsorption, ion exchange, charge transfer, and diffusion [77].

In other electrochemical techniques, quantitative analysis applying SWV is one of the most promising mechanisms in the production of biosensors, because of their ability to provide more sensitive answers for fast biosensing as compared to differential pulse voltammetry (DPV) techniques. In pulse methods, the systems depend on the usage of pulse changes of potential, and the usual response is measured at a suitable time relative to the time of the pulse [78].

Siuzdak et al. [79], worked on different methods for the diagnosis of pathogens. They applied a nanocrystalline boron-doped diamond-based electrode (B: NCD) as a platform for the biosensor.

Detection of avian influenza virus, (H5N1), is determined by different methods such as the immunochromatography, reverse-transcription PCR (RT-PCR), serological methods, ELISA [80], and fluorescence.

Moreover, the electrochemical diagnosis method gained much attention because it offered low costs, small specimen volume without an amplification step, and user-friendly interface and portability [81].

The hepatitis B e-antigen (HBeAg) immunosensor was also developed by applying electrochemical methods. Cocatalysis of nanoporous gold and horseradish peroxidase (HRP) were applied as modifier factors. The developed immunosensor demonstrated a linear realtionship between peak current and concentration of HBeAg (1 pg/mL to 1 ng/mL as well as 0.064 pg/mL of LoD (limit of detection)).

The electrochemical DNA biosensing instrument is an effective device due to properties, such as fast response time, user-friendly, high specificity, and sensitivity. Electrochemical paper analytical device (ePADs) creates a perfect contribution to the sensor, because of its use of the inexpensive paper substrate. Singhal et al. [82] reported the manufacturing of ePADs, by detecting the target DNA of the Chikungunya virus (CHIKV).

The most notable applied techniques include voltammetry, amperometry, and impedance spectroscopy methods. The most developed virus biosensors can be applied with integrated substrates for clinical, environmental, and industrial applications [83]. Some of the selected usages are listed in Table 1.

**Table 1:** Some selected studies on the detection of viruses by electrochemical methods

Target	Biosensor type	Nanomaterial	Sample type	Reference
Influenza virus M1 protein	Electrochemical impedance	Nanocrystalline boron-doped diamond	Saliva	[84]
Hepatitis C virus DNA	Electrochemical impedance	MB@SiNPs	Real patient specimen	[85]
Dengue virus DNA	Voltammetric	ZnO/Pt_Pd nanocomposite	–	[85]
HBV DNA	Electrochemical impedance	AuNPs	Real patient specimen	[86]
Avian influenza virus H5N1 gene	Voltammetric	MWCNTs_AuNPs	–	[87]
Dengue type 2 virus	Voltammetric	Nanoporous alumina	Infected Aedesaegypti Mosquito sample	[88]
Influenza virus	Voltammetric	CdS QDs	Real patient samples	[89]
Chikungunya virus DNA	Electrochemical paper analytical device	Gold shells_coated magnetic nanocubes	Serum	[90]
Influenza virus H1N1	Chronoamperometric	rGO	–	[91]

#### Aptamer-Based Detection

Aptamers are artificial single-stranded DNA or RNA oligonucleotides that have a high affinity towards a target, which they are designed to bind to [92]. Aptamer-based detection of viruses may be able to overcome barriers generated by the use of other methods such as costs and false-negative or false-positive results [93]. Aptamer-based biosensors or aptasensors, are fabricated by using aptamer as bioreceptors (capturing aptamer/probe) or transducers (signal aptamer/probe) [94]. In a comprehensive review by Zou et al. [95] on the application of aptamers in virus detection and antiviral therapy, a list of virus-specific aptamers that have been assessed but are yet to be used in the detection of viruses and treatment of viral infectious diseases, is introduced for the first time. Based on the transducer type, aptasensors are either electrical or optical.

##### Optical Aptasensors

1. Surface plasmon resonance or SPR aptasensors, which by evaluating the change of refractivity of a material bound on a surface, measures the resonance of free electrons in the metal films [96]. Typically, the capturing aptamer is immobilized on a metal surface. As the virus binds to the aptamer, the thickness of the metal surface changes, resulting in the alteration of the refractive index. Quantification can be carried out by monitoring the difference in the angle or intensity of light after the virus is bound to the aptamer. The advantages of this method are miniaturization, automation, and absence of labelling [97]. This method has been used for the detection of avian influenza virus (AIV) H5N1 [98, 99], and HIV-1 Tat protein [100].
2. Calorimetric-based aptasensors use a shift in color to detect a virus. This shift can be seen by the naked eye, or it will require a spectrophotometer. These types of aptasensors are cheap, simple, usually portable, and have been used in many applications [101]. They fall into different groups, for example, nanomaterial-based calorimetric aptasensors [102, 103] can use a nanomaterial as support for the aptamer or as a part of the transducer to create the signal with their assistance. Another type is the enzyme-linked aptamer assays (ELAA) which have the same base as ELISA, but use aptamers instead of antibodies as the bio-receptor or the transducer [104]. This type has been used to detect H5N1 [104], Zika virus [105], and others [106]. Lastly, aptamers have been used to modify lateral flow assay (LFA) for virus detection [107].
3. Fluorescent aptasensors, which can be categorized into aptasesnors that respond with fluorescent intensity [108], or the ones that respond with fluorescence polarization [109].
4. Others such as SERS based [110] and CL aptasensors [111].

##### Electrical Aptasensors

In this type, by binding of the aptamer to the target, production or change of an electrical signal occurs. Based on their detection mechanism they fall into two groups.

1. *Electrochemical aptasensors*

Usually in this type of sensor, the aptamer is immobilized on an electrode. In some applications, no enzyme is employed and binding of the immobilized aptamer to the target changes the impedance directly [112]. But in some others, the electrical signal change is assisted by employing enzymatic reactions [113]. The latter is a field-effect transistor (FET) which is a type of voltage-controlled semiconductor device that regulates electrical behavior by using an electric field and has been used to detect the Tat protein of HIV-1 [114].

2. *Piezoelectric Transducers*

Some materials such as the quartz crystal microbalance (QCM) have a Piezoelectric effect which is the ability to generate an electric charge in response to applied mechanical stress. In QCM aptasensors, the aptamer is fixated on the quartz crystal electrode so it can capture the target. The binding of the aptamer to the target changes the quality of the pole, which is subsequently converted into detectable frequency changes [115].

### Nanomechanical

B. Ilic et al. [116] used a resonating nanomechanical cantilever made from polycrystalline silicon to detect the immunospecific binding of viruses. Arrays of AcV1 antibody-coated polycrystalline silicon nanomechanical cantilever beams were used to detect binding at different concentrations of baculoviruses in a buffer solution. They calculated the mass of single-virus particles bound to the cantilever through a viable technique for sensitive detection of bound mass. Therefore, using this nanoelectromechanical device enables the selective detection of the virus through virus - antibody interaction (Table 2).

**Table 2:** Summary of the mentioned virus detection methods

Method	Advantages	limitations
**IF methods**	Inexpensive, accessibility, ease of operation, easy interpretation of results	Antisera concentration, the inability of the detection of new strains/species, instability of antibodies
**NAATs**	Ease of purification and separation of nucleic acids from antibodies, Excellent sensitivity, and specificity	Expensive, the technical expertise required
**Electron microscopy**	Accessible	The inability of the specific diagnosis of viruses
**Cell culture**	Capacity to isolate a wide variety of viruses, ease of operation	The long incubation period for some viruses,
**Aptamer- based**	Easy synthesize and modification of aptamers, a broad range of targets, inexpensive	Some methods need expert operators

## Applications of Viral Diagnostic Techniques for Different Types of Viruses

No single approach is optimal for detecting all viruses in all clinical situations. Therefore, it is vital to combine culture and nonculture methods to optimize viral disease diagnosis, yielding medically useful, cost-effective, and laborsaving viral testing results. In determining appropriate testing algorithms for the laboratory, one must consider a broad range of factors, including the patient population (i.e., age, immune level, etc.), clinical expressions, physician’s diagnosis, and time of year. However, with global travel and changing epidemiology of viral diseases, it isn’t possible to prepare a virology laboratory for unexpected epidemics or contamination [42].

### Testing for Respiratory Viruses

During periods of low virus activity, OIAs, or lateral-flow antigen assays may be used, but it is better to check all positive results via IF, viral culture, or RT-PCR [117]. Most of these assays have good specificity, which leads to a decrease in false-positives, hence achieving trustworthy results.

NAATs are another approach for respiratory virus detection. NAATs may be the only test, a supplemental test, or an additional test for specimen that obtain negative results by other test methods. The most reference laboratories perform NAAT testing for several virus diagnoses. Depending on the testing format, the costs will vary. In general, NAAT testing will be more costly than viral culture methods [118].

### Viral Testing of Vesicular Lesions

At first, direct fluorescent antibody (DFA) testing for VZV, HSV-1, and HSV-2 is carried out on direct smears of vesicular lesions. If the DFA is positive, virus isolation in cell culture is usually not useful, in contrast, if DFA is negative, cell culture-based methods should be used [42].

If NAAT testing is available, it should be considered due to its great sensitivity [119].

### Viral Testing of CSF (Cerebrospinal Fluid)

NAAT is more sensitive than culture techniques in detecting viral CNS pathogens [120]. The enteroviruses, the arboviruses, JC viruses, and the viruses in the *Herpesviridae* family (VZV, HSV-1, HSV-2, EBV, CMV, and HHV-6) are most commonly associated with CNS infections.

### Enteroviruses in CSF

Isolation of CSF enterovirus in cell culture is 75% less sensitive than NAATs. Therefore, NAATs are the most reliable method for the diagnosis of enterovirus infection in CSF [119]. 5_ UTR pan- enteroviral primers used in these approaches can detect a broad spectrum of enteroviruses, containing the nonculturable coxsackievirus strains. However, for the detection of parechoviruses, other probes are required [121].

### Viral Testing of Other Types of Samples

#### Fecal Samples for the Detection of Enteric Viruses

Detection of CMV, HSV, and enteroviruses can be carried out using cell culture methods. Adenovirus and rotavirus antigens can be recognized via EIA-based assays. Besides, NAATs or electron microscopy can detect noroviruses, astroviruses, and caliciviruses accurately [42].

#### Peripheral Blood Samples

Only a few types of viruses including CMV are isolated from peripheral blood. Rapid shell vial cultures of fibroblasts or H & V Mix yield sensitive results within 24 to 48 h or sooner. Although the detection of CMV in blood samples through shell vial culture shows less sensitivity when compared to CMV isolation in traditional tube cell cultures. However, it’s better to use tube cell cultures for samples with very low levels of virus.

Also, EBV, HHV-6, and/or BK viruses should be tested by NAATs. HSV and adenoviruses may also be isolated from blood cell cultures, rarely. NAATs also can be used for the detection of HSV and enteroviruses in blood from infants with neonatal sepsis [42].

#### Urine Samples

Detection of CMV and adenoviruses can be performed through shell vial and traditional tube cultures using a urine sample. In renal transplant patients, quantitation of the BK virus should be checked using NAATs [42].

## Methods Developed to Detect SARS-CoV-2

With the improvement in molecular biological techniques, nucleic acid detection methods have developed at a fast rate and are now considered as one of the best methods for virus detection [122–124]. At present, a suitable method for the diagnosis of new pneumonia in clinics and laboratories is chest radiographic findings, but the most accurate approach depends on nucleic acid-based assays [125]. However, the sample for nucleic acid assays from suspected patients of SARS-CoV-2 is mainly throat swabs [80]. This sample is not standardized and easily fails the detection process. The collection process of such samples is hazardous for medical personnel. The other method is RT-PCR, which is used for detecting and analyzing SARS-CoV-2 [126]. Polymerase chain reaction (PCR) has numerous advantages, such as fast diagnosis, high specificity, sensitivity, and this ordinary quantitative assay has been considered as the “gold standard” for virus detection [96]. The number of SARS-CoV-2-infected patients is increasing quickly, however, laboratory and clinical detection methods are limited [125].

Here below are a number of methods that researchers used for detection of the SARS-CoV-2 virus.

### PCR-Based Methods

As described above, PCR is an enzymatic method that creates multiple copies of a gene [69]. In general, SARS-CoV-2 involves mRNA converted into cDNA by reverse transcription, then PCR occurs and the resulting products undergo gel electrophoresis and sequencing to detect the SARS-CoV-2 virus [70, 71].

Corman et al. [72], used respiratory specimens from infected cases at Charité Medical Center during 2019. They obtained cell culture supernatants from SARS-CoV-2 in their clinical laboratories, and extracted their RNA for PCR purposes.

They used all complete sequences of SARS-related viruses that were available at GenBank by January 1st, 2020 to create oligonucleotides and carry out in-silico evaluation. Then, they used these sequences for alignment and assay design.

**Fig. 2:**
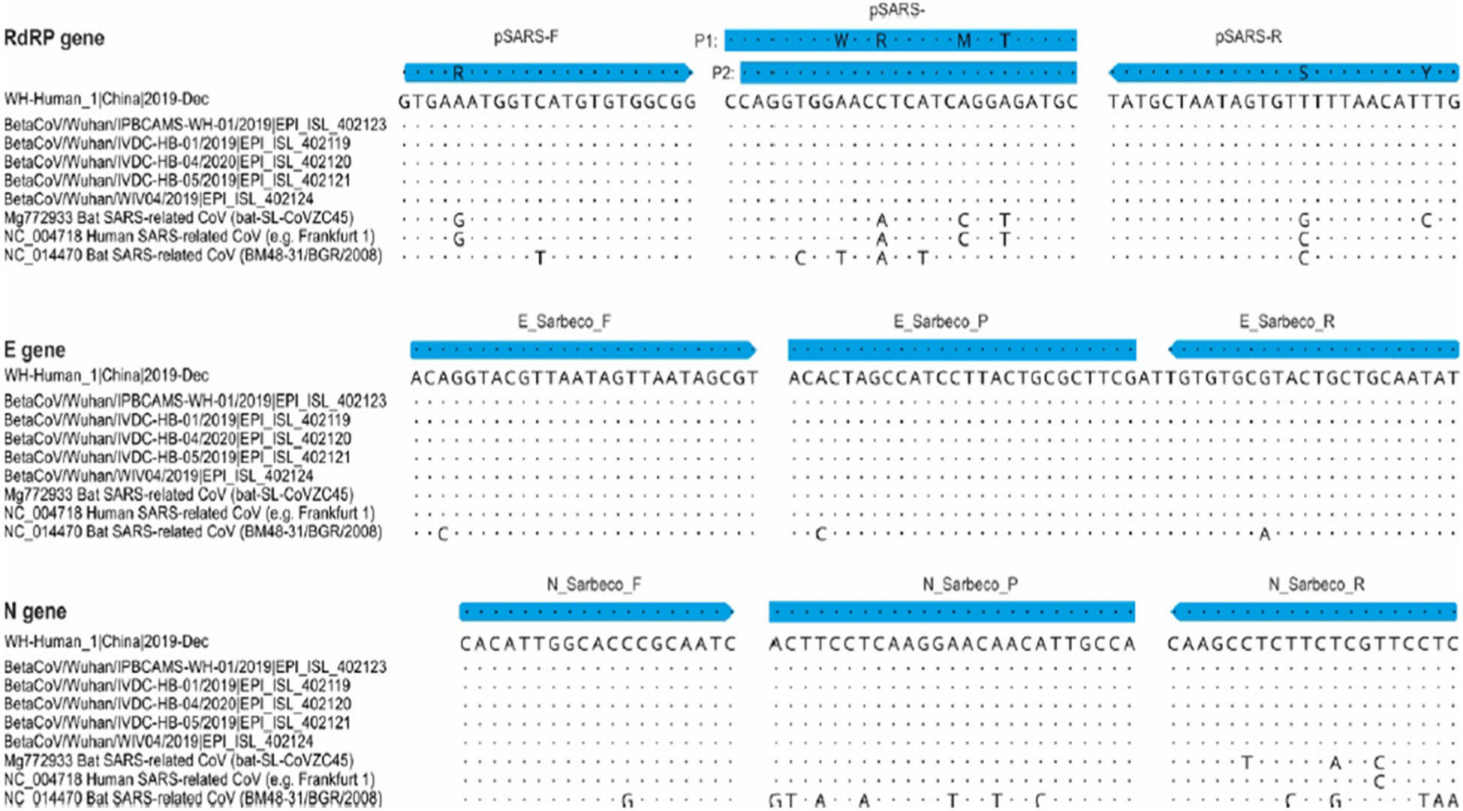
Partial alignments of oligonucleotide binding regions. Panels show six available sequences of the Wuhan-CoV, aligned to the corresponding partial sequences of SARS-CoV strain Frankfurt 1, which can be used as a positive control for all three RT-PCR assays. The alignment also contains the most closely-related bat virus (Bat SARS-related CoV isolate, bat-SL-CoVZC45, GenBank Acc.No. MG772933.1) as well as the most distant member within the SARS-related bat CoV clade, detected in Bulgaria (GenBank Acc. No. NC_014470). Dots represent identical nucleotides compared to Wuhan-Hu 1. Substitutions are specified. More comprehensive alignments in the Appendix. Reprinted with permission from ref. [127]

In the end, they performed sequence alignments with those from the Wuhan cluster. All of the sequences that they found matched the amplicons (Fig. 2).

In a study by Corman et al. [127], they indicate the most closely-related bat virus and the distant member within the SARS-related bat CoV clade to Wuhan-Hu 1. They show that in all assays researchers can use SARS-CoV genomic RNA as a positive control.

In another study, real-time reverse-transcription PCR assays for detecting the different parts (ORF1b and N) of the viral genome were carried out. Accordingly, the probe and primer were designed in such a way that they could react to this new SARS-CoV-2 and other SARS-CoV viruses including SARS and MERS [71].

They used two different kits; for RNA extractions, they used the viral RNA purification kit (QIAamp Viral RNA Mini Kit, Qiagen), and for DNA extractions they used the DNA plasmid purification kit (QIAprep Spin Miniprep Kit, Qiagen).

Moreover, they obtained respiratory samples from two SARS-CoV-2-infected cases. For the first patient, a sputum sample was obtained on day 5 after the first appearance of symptoms. For the second patient, a throat swab sample was obtained on day 3 after the symptoms appeared. The samples were then stored in standard virus transport media.

They utilized RNA from cells that were infected with SARS-CoV-2 as a positive control. Furthermore, after RT-PCR, they cloned the products of the SARS-CoV-2 formed by the ORF1b and N gene assays into plasmids.

In this study, researchers used two assays with different viruses, due to insufficient information about this novel SARS-CoV-2 virus in humans and animals.

They achieved some interesting data, which showed that, when RNA from infected cells is used as a positive control, assays indicate a range of seven orders of (2*10^− 4^-2000 TCID50/reaction), and when DNA plasmid was used as a positive control, then the diagnoses of the assays were below 10 copies for each reaction. The specimen from the infected cases were positive in the test.

They claimed that these assays can quickly detect this novel SARS-CoV-2 in human specimens, and this will help to identify patients as soon as possible.

### Enzyme-Linked Immunosorbent Assay (ELISA)

Xiang et al. [123] confirmed two new kits for detecting the novel SARS-CoV-2 virus. These kits have acquired significant results, but they are not available on the market. In the above study, all the SARS-CoV-2-infected cases at the Jinyintan Hospital, starting from January 1 to January 28, 2020, were investigated.

The new IgG/IgM antibody enzyme-linked immunosorbent assay (ELISA) kit was obtained from the Zhuhai Livzon Diagnostics Inc., China. Serum specimens for the ELISA IgG and IgM antibodies tests were obtained from infected cases on February 2, 2020. The 98 serum specimens were tested in accordance with the manufacturer’s instructions.

Results of the ELISA test for the 63 samples of SARS-CoV-2-infected cases indicates that 28 IgM antibodies were positive, the accuracy of the test was 82.54% (52/63) with specificity of 100% (35/35) and 52 IgG antibodies were positive, the accuracy of the test was 88.8% (87/98) with the specificity of 100% (35/35), and the sensitivity for the detection of the combined IgM and IgG was 55/63 (87.3%) and the healthy controls was negative.

#### Colloidal Gold-Immunochromatographic Assay (GICA)

A new IgG/IgM antibody colloidal gold-immunochromatographic assay (GICA) kit for detecting SARS-CoV-2 was obtained from the Zhuhai Livzon Diagnostics Inc., China. Ninety one plasma specimens for the GICA were obtained from infected cases on February 3–4, 2020. The 126 plasma specimens were tested as per the manufacturer’s instructions.

Result of the ELISA test for the 91 samples of COVID-19-infected cases indicated that 52 IgM antibodies were positive, the accuracy of the test was 69.0% (87/126) with the specificity of 100% (35/35), and 74 IgG antibodies were positive, the accuracy of the test was 86.5% (109/126) with the specificity of 100% (35/35. The sensitivity for the detection of combined IgM and IgG was 82.4% (75/91) and the healthy controls was negative [126].

#### qRT-PCR Assay for SARS-CoV-2

Three different samples including a sputum specimen, throat swab specimen, and alveolar lavage fluid samples were obtained from suspected cases of SARS-CoV-2 infection. The goal was to extract the RNA from these samples using the nucleic acid extraction kit (QIAamp viral RNA mini kit). The qRT-PCR was carried out using the ABI 7500 Real-Time PCR system. Two target genes, including the open reading frame 1ab (ORF1ab) and nucleocapsid protein (N), were setup together and tested during the qRT-PCR assay. This procedure used a SARS-CoV-2 nucleic acid detection kit, as per the manufacturer’s instructions (Shanghai ZJ Bio-Tech Co., Ltd). The result of the qRT-PCR test for 81 samples of SARS-COV-2-infected cases indicated that 42 cases were positive and the sensitivity was 51.9% (42/81). They compared the sensitivity of the ELISA IgM+ IgG, GICA IgM + IgG, and qRT-PCR methods and found significant variances between these methods (*P* < 0.001). However, there is no significant variance between the sensitivity of ELISA (IgM + IgG) and GICA (IgM+ IgG), *P* = 0.411 [126].

In another study, Amanat et al. [74] found a new way of detecting SARS-COV-2 by using recombinant antigens extracted from the spike protein of SARS-CoV-2. The method depends on reactivity to the immunogenic S protein of the virus. These assays were formed with a negative control specimen showing pre-SARS-CoV-2 background immunity in the general population and samples obtained from the SARS-COV-2 infected cases.

They produced two different versions of the spike protein. The first structure declares a complete trimeric and stabilized version of the spike protein and the second one expresses only the much smaller receptor-binding domain (RBD). The sequence applied for both proteins depends on the genomic sequence of the first virus isolate, Wuhan-Hu-1, which was extracted on January 10th, 2020.

The plasma/sera sample were obtained from infected cases at day 20 (SARS-CoV2#1), on day 4 (SARS-CoV-2 #2), days 2 and 6 (SARS-CoV-2 #3A and B), after the symptoms appeared.

They applied a panel of 59 banked human serum specimens obtained from candidates who had previously-confirmed viral infections (e.g., hantavirus, dengue virus, SARS-CoV-2 NL63 – specimen collected 30 days after the first symptom appeared) to establish an ELISA with these proteins.

ELISAs were conducted by doing serial dilutions of the individual serum specimens. All COVID-19 plasma/serum specimens reacted strongly to both RBD and the full-length spike protein, as the reactivity of the other serum specimens only yielded background reactivity.

Generally, the reactivity of SARS-CoV-2 sera was better against the full-length S protein than against the RBD. This may happen due to the higher number of epitopes existing on the much larger spike protein [126].

#### Antibody Isotyping and Subtyping

For the four COVID-19-infected plasma/sera cases, Amanat et al. [74] also performed an isotyping and subtyping ELISA, which applied the mammalian cell and insect cell-expressed S proteins. Reactivity for all specimens for IgG3, IgM, and IgA was strong.

An IgG1 signal was also discovered for three out of the four specimens, while one sample indicated no reactivity. There was not any signal for IgG4 and reagents for IgG2 were not available.

The results indicate that these assays are sensitive and specific, allowing for diagnosis of SARS-COV-2, by applying human plasma/serum as soon as 3 days after the symptoms appear. Most importantly, these methods do not need handling of infectious viruses and they can be modified to detect different kinds of antibodies. They claimed that their ELISA method will play a key role in determining the real rate of attack and rate of infection fatality in different human populations, and also in designing the kinetics of the antibody response to SARS-CoV-2 [127].

### Microarray-Based Method

The microarray-based method is a quick diagnostic procedure. In this method, at first RNA from the SARS-CoV-2 virus produces cDNA which is labeled with special probes during the reverse transcription stage. Afterward, these labeled cDNAs will be loaded onto the solid phase microarrays. Oligonucleotides are fixed on it, which will then go through a series of washing steps, and the free DNAs will be subsequently deleted.

In the end, with the special diagnosis probes, SARS-CoV-2 RNA will be detected. Because of its advantages, this method is one of the popular techniques for the diagnosis of COVID-19 [75].

In one study, Shi et al. [76] detected the SARS-CoV-2 virus in a clinical specimen by developing a 60-mer oligonucleotide microarray, based on the TOR2 sequence.

Guo et al. [77] designed a microarray to diagnose 24 single nucleotide polymorphism (SNP) mutations across the spike (S) gene of SARS-CoV, and the accuracy of the method was 100% with regard to the detection of the SARS-CoV-infected specimen.

In another study, Luna et al. [78] developed a nonfluorescent oligonucleotide array for diagnosis of the entire SARS-CoV genus. The advantage of this microarray was its low costs and high sensitivity when compared to real-time RT-PCR.

### Newly Developed Methods

One of the novel methods for detecting RNA is the RNA-targeting clustered regularly interspaced short palindromic repeats (CRISPR) method which makes use of the enzyme, Cas13 [81, 82].

Zhang et al. [128], indicated that Cas13 can be used to target and destroy the different mammalian single-stranded RNA viruses. They produced a platform named SHERLOCK, which uses isothermal pre-amplification along with Cas13 to detect DNA or RNA. Their new protocol for SARS-COV-2, named “A protocol for detection of SARS-COV-2 using CRISPR diagnostics”, has been introduced on the website (https://broad.io/sherlockprotocol), this protocol may help researchers to detect nucleic acids.

### Immunosensor

Layqah and Eissa managed to fabricate an Immunosensor for detecting the SARS-CoV-2 virus, based on the array of gold nanoparticle-modified carbon electrodes [129]. The carbon electrode chip endured surface modification using AuNPs, so that the electron transfer rate and surface area would increase. In this work, AuNPs were electrodeposited on carbon disposable array electrodes; this was then used for electrode preparation. Such an immunosensor can be used for spiked nasal samples to simultaneously detect different types of CoV virus. This device was made out of eight electrodes, attached to the same chip. These electrodes were covered with human SARS-CoV-2 and MERS-CoV antigens. Two of the electrodes were bound to BSA instead of antigens for comparative purposes and to be used as controls. Also for each antigen, two electrodes were modified so the results could be more reliable.

The principle of the method is that if an antibody is present in the sample, it binds to the antigens immobilized on the surface of the electrodes. When power current is running through the sample, if an antibody is bound on the electrodes, the current will decrease. This is because the antibody is bulky and will prevent electrons from moving further. A fixed concentration of antibody was applied, so as to be compared to the results of the specimens. This device showed high sensitivity, as the detection range for MERS-CoV and SARS-CoV-2 were 0.001–100 and 0.01–10,000 ng/ml^− 1^, respectively. Also, it showed to be highly selective to MERS-CoV and SARS-CoV-2 antibodies among others such as Flu A and Flu B. The applications of gold nanoparticles for virus detection have been discussed fully in a review by Draz et al. [130].

## Samples

Rapid collection and testing of appropriate samples collected from suspicious patients is a necessary step for management and control of a SARS-CoV-2. Suspected cases should be screened for the genome of the virus using nucleic acid amplification tests (NAAT), such as RT-PCR.

### Categories of Specimen [131]

1. Upper respiratory tract specimens: These include nasopharyngeal and pharyngeal swabs.
2. Lower respiratory tract specimens: These include deep-cough sputum, alveolar lavage fluids, bronchial lavage fluid, and respiratory tract extracts.
3. Fecal specimens: Fecal samples are about 10 g in weight.
4. Blood specimens: One should, as much as possible, collect anticoagulated blood in the acute phase within 7 days after the onset of the disease. A 5 ml sample of blood is required for each collection.

Vacuum tubes containing EDTA anticoagulants are recommended for blood collection.

5) Serum specimens: Both acute-phase and convalescent serum specimens should be collected as much as possible. The first serum specimen should be collected as soon as possible (preferably within 7 days after the onset of illness), and the second specimen should be collected during 3–4 weeks after the onset of illness. A 5 ml sample of blood is required for each specimen. Serum specimens are mainly used for measuring antibodies, rather than nucleic acid testing.

### Methods of Specimen Collection and Processing [132, 133]

1. Nasopharyngeal swab: The sampler gently holds the person’s head with one hand, the swab in
  another, insert the swab via nostril to enter, slowly get deep along the bottom of the lower nasal
  canal. Because the nasal canal is curved, do not force too hard to avoid traumatic bleeding. When
  the tip of the swab reaches the posterior wall of the nasopharyngeal cavity, rotate gently once, then slowly remove the swab and dip the swab tip into a tube containing 2-3 ml virus preservation solution (or isotonic saline solution, tissue culture solution or phosphate buffer), discard the tail, and tighten the cap.
2. Pharyngeal swab: The sampled person first gargles with normal saline, the sampler immerses the swabs in sterile saline (virus preservation solution is not allowed to avoid antibiotic allergies), holds the head of the sampled person up slightly, with one’ s mouth wide open, making a sound “ah” to expose the lateral pharyngeal tonsils, insert the swabs, stick across the tongue roots, and wipe both sides of the pharyngeal tonsils with pressure at least 3 times, then wipe on the upper and lower walls of the pharynx for at least 3 times, and dip the swabs in a tube containing 2–3 ml storage solution (or isotonic saline solution, tissue culture solution or phosphate buffer solution), discard the tail and tighten the cap. The pharyngeal swabs can also be placed in the same tube together with the nasopharyngeal swab.
3. Nasopharyngeal or respiratory tract extract: Extract mucus from the nasopharynx or extract
  respiratory secretions from the trachea with a collector connected to a negative-pressure pump;
  insert the head of the collector into the nasal cavity or trachea, turn on the negative pressure, rotate and slowly withdraw the head of the collector, collect the extracted mucus, and rinse the collector once with 3 ml of sampling solution.
4. Deep cough sputum: Ask the patient to cough deeply, and collect the sputum coughed up in a 50 ml screw-capped plastic tube containing 3 ml of sampling solution. If the sputum is not collected in the sampling solution, 2–3 ml of the sampling solution can be added into the tube before testing, or add sputum digestive reagents of an equal volume of sputum.
5. Bronchial lavage fluid: Insert the head of the collector into the trachea (about 30 cm deep) from the nostril or the tracheal insertion part, inject 5 ml of physiological saline, turn on the negative pressure, rotate the head of the collector and slowly withdraw it. Collect the extracted mucus and rinse the collector once with the sampling solution (a pediatric catheter connected to a 50-ml syringe may be used as an alternative to the collector).
6. Alveolar lavage fluid: After local anesthesia, insert a bronchoscope through the mouth or nose, pass through the pharynx into the branch of the right middle lobe or the lingular segment of the left lung, and insert the tip into the bronchial branch opening; slowly add sterilized physiological saline through the biopsy hole of the bronchoscope, with 30–50 ml of saline each time, 100–250 ml in total, 300 ml at most.
7. Fecal specimen: Take 1 ml sample treatment solution, pick up a little sample about the size of soybean and add it into the tube, gently blow for 3–5 times, set aside at room temperature for 10 min, centrifuged at 8000 rpm for 5 min, absorb the supernatant for detection. Fecal specimen treatment solution can be prepared in-house by the laboratory: 1.211 g tris, 8.5 g sodium chloride, 1.1 g calcium chloride anhydrous or 1.47 g calcium chloride-containing crystalline water, dissolved into 800 ml of deionized water, with the pH adjusted to 7.5 with concentrated hydrochloric acid and replenishing with deionized water to 1000 ml. Stool suspensions can also be prepared using HANK’s solution or other isotonic saline solution, tissue culture solution, or phosphate buffer solution. If the patient has diarrhea symptoms, collect 3–5 ml of stool specimen, gently blow and mix, centrifuge it at 8000 rpm for 5 min, absorb the supernatant to reserve for use.
8. Anal swab: Gently insert the disinfectant cotton swab into the anus for 3-5 cm in-depth, then gently rotate and pull out, immediately put the swab into a 15-ml screw-capped sampling tube containing 3–5 ml of virus preservation solution, discard the tail and tighten the tube cover.
9. Blood samples: It is recommended to use vacuum blood vessels containing the EDTA anticoagulant to collect 5 ml of blood samples. Nucleic acid extraction should be performed on whole blood or plasma according to the type of nucleic acid extraction reagent selected. For plasma separation, the whole blood should be centrifuged at 1500 to 2000 rpm for 10 min, and the supernatant will be collected in sterile plastic tubes with a screw cap.
10. Serum specimen: Collect a 5-ml blood specimen with a vacuum negative-pressure blood collection tube. Keep the specimen at room temperature for 30 min, centrifuge it at 1500–2000 rpm for 10 min, and collect the serum in a sterile plastic tube with a screw cap (www.chinacdc.cn).

Other samples that can be collected for detecting SARS-CoV-2 are blood and stool [134–138] (Table 3).

**Table 3:** Types of specimen, collection material, and storage

Specimen type	Collection materials	Storage temperature until testing in-country laboratory	Recommended temperature for shipment according to expected shipment time
Nasopharyngeal and oropharyngeal swab	Dacron or polyester flockedswabs	2–8 °C	2–8 °C if ≤5 days –70 °C (dry ice) if > 5 days
Bronchoalveolar lavage	Sterile container	2–8 °C	2–8 °C if ≤2 days –70 °C (dry ice) if > 2 days
(Endo) tracheal aspirate, nasopharyngeal or nasal wash/aspirate	Sterile container	2–8 °C	2–8 °C if ≤2 days –70 °C (dry ice) if > 2 days
Sputum	Sterile container	2–8 °C	2–8 °C if ≤2 days –70 °C (dry ice) if > 2 days
Tissue from biopsy or autopsy including from lung.	Sterile container with saline orVTM	2–8 °C	2–8 °C if ≤2 days –70 °C (dry ice) if > 2 days
Serum	Serum separator tubes (adults:collect 3–5 ml whole blood).	2–8 °C	2–8 °C if ≤2 days –70 °C (dry ice) if > 2 days
Whole blood	Collection tube	2–8 °C	2–8 °C if ≤2 days –70 °C (dry ice) if > 2 days
Stool	Stool container	2–8 °C	2–8 °C if ≤2 days –70 °C (dry ice) if > 2 days
Urine	Urine collection container	2–8 °C	2–8 °C if ≤2 days–70 °C (dry ice) if > 2 days

## Future of Diagnostic Methods for Viral Infections

Tissue culture is considered as the gold standard for viral diagnosis [139], but in the case of epidemics such a SARS-CoV-2, this method has the disadvantage of being time-consuming. Hence when rapid diagnosis is required, the tissue culture technique is rarely considered. However, when it comes to research, this method is the best one for detection and characterization of viruses isolated from cells and investigating the response of each host to viral infection [139].

Recently, Kaya et al. [83] reviewed the progress in electrochemical methods for the diagnosis of viral infections. These methods can be designed and specified for different viruses. They are fast, not expensive, and user-friendly. However, they must be modified to be suitable for a medical diagnostic laboratory, so as to compete with the currently most used methods, like RT-PCR. Most importantly, they should be as sensitive as molecular methods, such as RT-PCR.

A systematic review carried out by Pang et al. [140] indicated that molecular tests such as RT-PCR show higher sensitivity and specificity than serological ones, such as ELISA. However, Xiang et al. [141] in a recent study on COVID-19 patients showed that while using ELISA and GICA kits designed for IgG/IgM antibodies and serum samples, sensitivity was high enough to compete with the qRT-PCR method. This can be promising with regard to modifying serological methods for viral infection diagnosis, as they can get sensitive enough to replace PCR-based methods. Moreover, they are less time-consuming than PCR and don’t have the risk of showing false results, because of being contaminated [83]. Other methods based on antibody recognition, which were also discussed previously in this paper, can be adapted for different viruses if they could show enough sensitivity as well.

Microarray-based methods including the ones discussed here, have shown adequate sensitivity, almost as high as the RT-PCR method, and considering that they are cheaper and less time-consuming than RT-PCR, they can be very promising in the development of a new clinical diagnostic method.

As for SARS-CoV-2, WHO advises laboratories that while encountering a suspected case, nucleic acid amplification tests (NAAT), such as RT-PCR, should be carried out for virus detection. The world health Organization (WHO) has also presented several instructions regarding specimen collection. In fact in a recent investigation by Gu et al. [142], stool specimens were confirmed to contain the virus, even in patients that were discharged. So the WHO advises that stool specimens should be taken from suspects, as well as respiratory samples [1]. Nucleic acid amplification tests (NAAT), are the only methods confirmed by WHO for diagnosing SARS-CoV-2 infections, so any proposed method should show sensitivity as high as that of the RT-PCR method. Furthermore, it also be more affordable and faster than the current available molecular methods.

## Conclusion

COVID-19 is an ongoing pandemic disease, and since the progress of infection by the SARS-CoV-2 virus can lead to severe permanent respiratory problems and possible death, there is an urgent need to provide various diagnostic strategies for early detection of the disease. SARS-CoV-2 is a novel coronavirus, therefore, it is not yet possible to determine with certainty what method is the best for reliable and accurate diagnosis of the disease.

Although the clinical symptoms of COVID-19, such as fever, cough, dyspnea, acute kidney injury, liver damage may help healthcare workers make a primary diagnosis, but the final detection of the COVID-19 in patients is confirmed by one of the approved methods that include lung CT scans, molecular assays, and serological tests. Moreover, nowadays, several other virus detection methods are available for viral diseases, the use of which depend on specific properties of each virus or virus family, however, they are not routine and specific for COVID-19 detection, which requires further research for the development of more accurate tests. Besides the type of sample and sampling procedures, clinical specimens are also of critical importance in acquiring the most reliable test results for COVID-19. In conclusion, the understanding of the advantages and disadvantages of various virus detection techniques and their proper implementation could prevent further virus outbreaks, provide efficient control of the current pandemic situation, and prevent medical staff fatigue, all of which will ultimately help in the promotion of public health and safety.

Hence, researchers should focus more on different approaches, in order to ultimately find a highly efficient and accurate method for the rapid and precise detection of viral diseases like COVID-19. In this way, the devastating outcomes of the disease could be prevented.

## Data Availability

Not applicable for this study.

## Abbreviations

SARS-CoV-2: Severe acute respiratory syndrome coronavirus 2
PHEIC: Public health emergency of international concern
MERS: Middle East Respiratory Syndrome
SARS: Severe Acute Respiratory Syndrome
RT-PCR: Reverse-transcription Polymerase Chain Reaction
rRT-PCR: Real-time RT-PCR
CT: Computed tomography
RNA: Ribonucleic Acid
DNA: Deoxyribonucleic Acid
IFA: Indirect Fluorescent-Antibody
EIAs: Enzyme Immunoassays
ELISA: Enzyme-Linked Immunosorbent Assay
NAATs: Nucleic Acid Amplification Tests
PCR: Polymerase Chain Reaction
HDA: Helicase Dependent Amplification
RPA: Recombinase Polymerase Amplification
LAMP: Loop-Mediated Isothermal Amplification
NGS: Next-Generation Sequencing
NEMS: Nanoelectromechanical Systems
NPs: Nanoparticles
NMs: Nanomaterials
EIS: Electrochemical Impedance Spectroscopy
SWV: Square Wave Voltammetry
DPV: Differential Pulse Voltammetry
B: NCD: Boron-doped Nanocrystalline Diamond-based
HBeAg: Hepatitis B e Antigen
CHIKV: Chikungunya Virus
ELAA: Enzyme-linked Aptamer Assays
FET: Field-effect Transistor
QCM: Quartz Crystal Microbalance
WHO: World Health Organization
